# Non-Targeted Screening and Identification of the Transformation Pathway of Carbamazepine in the Saemangeum Watershed, Republic of Korea

**DOI:** 10.3390/ijms252211947

**Published:** 2024-11-07

**Authors:** Da Rae Jeon, Young-Eun Kim, Jong Kwon Im, Yujeong Huh, Hyoung Seop Kim

**Affiliations:** Environmental Measurement & Analysis Center, National Institute of Environmental Research, 42 Hwangyong-ro, Incheon 22689, Republic of Korea; ekfo1228@korea.kr (D.R.J.); happyday23@korea.kr (Y.-E.K.); lim-jkjk@daum.net (J.K.I.); huhyujeong@korea.kr (Y.H.)

**Keywords:** carbamazepine, transformation products, LC–Orbitrap, Saemangeum, non-target

## Abstract

Carbamazepine (CBZ) is a widely used pharmaceutical for various purposes, including as an anticonvulsant, antibiotic, and antiepileptic agent, and it undergoes diverse metabolic pathways in both the environment and the human body. Therefore, this study aimed to explore the distribution of CBZ, the presence of its transformation products (TPs), and the transformation pathways in the Mangyeong and Dongjin Rivers in the Saemangeum watershed of Korea using non-targeted screening. The concentration distribution results for CBZ and its TPs showed that the average concentrations in the Mangyeong and Dongjin Rivers were 128.8 ng/L and 89.0 ng/L, respectively. The Mangyeong River exhibited a higher CBZ concentration than the Dongjin River, which was similar to those of the reported CBZ concentrations in other major domestic and international rivers. The types and detection frequencies of the identified TPs exhibited similar trends. The detection frequencies of the TPs decreased in the following order: CBZ-EP > DiOH-CBZ > 10OH-CBZ > 2OH-CBZ > 9-carboxyacridine > 9-acridinecarboxaldehyde. The detection frequency of the main TPs was high, and some were believed to be generated during the water treatment process. The presence of additional TPs (CBZ-O-quinone, acridine, and iminostilbene) was confirmed by the generated molecular networks. This study presents the transformation pathway of the CBZ and provides foundational data for understanding the environmental behavior of TPs, improving wastewater treatment plants, managing water quality, and establishing water environmental policies.

## 1. Introduction

With the increasing use of organic substances, the production of various mixtures has also increased. Artificially created compounds are discharged into water systems through sewage and wastewater treatment facilities and potentially affect both human and aquatic ecosystems. Various water-treatment processes have been developed and implemented to remove these pollutants; however, technological limitations remain in the elimination of pollutants with varying physicochemical properties [1,2].

Many countries globally, including South Korea, define potential pollutants that can cause environmental issues as “emerging contaminants (ECs)” and implement policies focusing on their identification, monitoring, and discharge management. ECs include pharmaceuticals, personal care products, cosmetics, industrial chemicals, and pesticides [3,4]. Carbamazepine (CBZ) belongs to the class of antiepileptic drugs (AEDs) and is one of the oldest drugs used for the treatment of epilepsy. It undergoes various transformation processes in the human body, leading to the formation of multiple transformation products (TPs) [5,6]. CBZ is primarily transformed in the liver and excreted through urine or feces, and dozens of TPs were identified [7,8,9]. Generally, TPs exhibit higher hydrophilicity and lower toxicity than that of their parent compounds, although in some cases, they may demonstrate higher toxicity [10,11]. For example, certain transformation products (DiOH-CBZ, 2-OH-CBZ, and 3-OH-CBZ) of CBZ were reported to exhibit higher chronic toxicity in freshwater organisms than CBZ itself [11]. CBZ is a pharmaceutical substance with a long half-life and is not easily biodegradable in aquatic environments. It is used as an indicator of water pollution because it does not readily decompose in wastewater treatment plants (WWTPs) [1,12,13]. Various studies have attempted to decompose CBZ in WWTPs using chlorine, ultraviolet light, and other methods; however, it was reported that, in some cases, the concentrations of CBZ and certain TPs increase after wastewater treatment [14,15,16]. CBZ and its TPs enter sewage systems through human activities and exist at low concentrations in surface water. However, they also exhibit biological activity and can have specific effects on humans and other organisms. Consequently, the exposure of these substances to the environment can potentially induce toxicity, which has garnered significant attention from the global scientific community [17,18,19]. Research on the derivatives of CBZ is ongoing, and studies have been conducted to investigate the presence and toxicity of CBZ and its TPs in three rivers in France (the Thouet, the Clain, and the Vienne Rivers) using various treatment methods such as ozone treatment and electrochemical oxidation [18,20,21].

Identifying TPs in terms of water quality is the first step in revealing the transformation pathways and major conversion reactions in WWTP and aquatic environments. The analysis method that can identify unknown compounds in complex environmental matrices is called non-targeted screening (NTS) [22,23]. Non-targeted analysis (NTA), a subset of NTS, involves real-time comparison of mass analysis information with an online web-based database (DB) to explore the detected contaminants. Studies were conducted using full-scan data obtained using NTA. NTA combines time-of-flight (QTOF) and Orbitrap mass analyzers to acquire MS and MS^2^ data across a wide *m*/*z* range, enabling the comprehensive analysis of chemical substances in the sample [24,25,26]. Molecular networking is currently used as an NTS method in TP analysis. This tool is used for exploring novel substances by identifying molecular families through the structural analysis of mass spectrometry data. Assuming that molecular families exhibit similar fragmentation patterns, the similarity between spectra can be calculated using the cosine score and visualized in the form of a network with nodes and edges. Non-targeted analysis is widely utilized in various fields, such as food chemistry and environmental science, in addition to bioinformatics, due to its ability to explore new substances through structural analysis [27,28,29]. The software Compound Discoverer 3.3 has a feature called the “Generate Molecular Networks node (GMN)”, which can be utilized for similar purposes. It is used as a tool for TP identification by processing data and searching for TPs [30,31].

The objective of this study was to apply non-targeted analysis using LC–Orbitrap MS and Compound Discoverer 3.3 to attain the following targets: (1) determining the concentrations of CBZ in the Mangyeong and Dongjin Rivers and identifying TPs using non-targeted analysis and GMN; (2) assessing the spatial and temporal distribution of detection; and (3) proposing a transformation pathway for CBZ.

## 2. Results and Discussion

### 2.1. Carbamazepine Concentration in the Mangyeong and Dongjin Rivers

This study analyzed the concentration and detection frequency of CBZ in samples collected once a month for one year from three selected locations in the Mangyeong and Dongjin Rivers. Detection frequency refers to the percentage of which CBZ was detected above the LOQ (40.6 ng/L). It is calculated as the number of samples where CBZ was detected divided by the total number of samples collected at each site, expressed as a percentage. The average detection frequencies at the Mangyeong River sites (M1, M2, and M3) were above 41.7% with concentrations ranging from 43.8 to 288.2 ng/L. At the Dongjin River sites (D1, D2, and D3), the average detection frequency was 13.9%, and the concentrations ranged from 51.7 to 177.0 ng/L. The ranges, means, medians, and DF% values for each location are shown in Table 1, where n/d refers to samples where CBZ was not detected; n = 12 indicates the number of monthly samples collected over one year at each site; and n = 36 represents the total number of samples collected across all sites. Similar CBZ concentrations were reported in other major rivers in South Korea. Concentrations ranging from 0.5 to 1763.0 ng/L [32] were reported in the Yeongsan River, from 25.0 to 480.0 ng/L [33] in the Nakdong River, from 3.7 to 65.5 ng/L [14] in the Han River, and from 2.0 to 65.0 ng/L [34] in the tributaries in the Geum River. In other countries, concentrations of 30.4–272.6 ng/L in the Danube River in Hungary [35], 92.2–136.0 ng/L in the Liobregat and Besòs Rivers in Barcelona [36], 2.0–383.0 ng/L in the Yodo River in Japan [37], and 280.0–740.0 ng/L in the St. Lawrence River in Canada [38] were detected. Evidence from domestic and international studies demonstrates that similar or higher concentrations of CBZ, comparable to that of our values, are detected in surface water.

### 2.2. Analyzing TPs of Carbamazepine Using Non-Targeted Analysis

**Figure 1:**
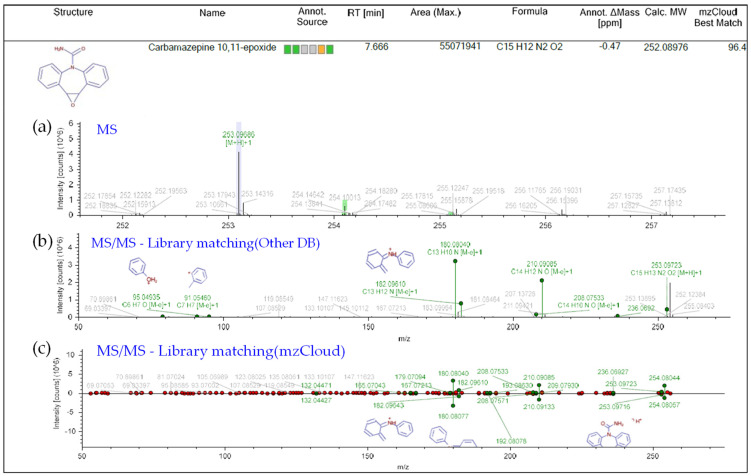
Overview of compound identification workflow by Compound Discoverer3.3 showing the Level 2 identification of EP-CBZ in sample as an example: (**a**) MS spectrum (**b**) Level 2b: MS/MS spectrum matching—other DB (**c**) Level 2a: MS/MS spectrum matching—mzCloud.

Six and three types of CBZ TPs were detected at two locations on the Mangyeong River (M2 and M3) and one location on the Dongjin River (D3), respectively. The names, molecular formulas, and mass error ranges of the detected TPs were verified, and information such as the formula, *m*/*z*, structure, MS, and MS^2^ patterns are included in Figure 1 and Table 2. Therefore, the rules proposed by Schymanski et al. [39] were referred to for verifying the confidence level. Detected TPs had a confidence level 2, and among them, those with MS and MS/MS data—such as carbamazepine 10,11-epoxide—and those with MS/MS patterns matching other databases, as shown in Figure 1b, were assigned a confidence level 2b. If matching with the instrument library (mzCloud) was successful, as shown in Figure 1c, a confidence Level 2a was assigned. Information on the other TPs is provided in Figure S1 and the definition of the confidence levels is explained in detail in Section 3.5 and Table 3. The TPs detected in both the Mangyeong and Dongjin Rivers were 10,11-dihydro-10,11-dihydroxycarbamazepine (DiOH-CBZ), carbamazepine 10,11-epoxide (CBZ-EP), and 10,11-dihydro-10-hydroxycarbamazepine (10OH-CBZ). These substances are well-known TPs of CBZ and all have a confidence level 2a [8,40,41]. They are primarily transformed in the liver through three main transformation pathways but are also degraded by ozonation and oxidation in some WWTPs [18,20,21]. In the human body, the primary transformation pathway involves the conversion of CBZ to CBZ-EP by cytochrome P450 (CYP450), followed by the formation of DiOH-CBZ through a hydration process. The second pathway involves the sequential oxidation of carbamazepine by CYP1A2, resulting in the formation of 2-hydroxycarbamazepine (2OH-CBZ) and 10OH-CBZ [8,40,41]. Substances detected only in the Mangyeong River were 2OH-CBZ, 9-acridinecarboxaldehyde, and 9-carboxyacridine, with 2OH-CBZ being the TP produced via the second transformation pathway. Intermediate TPs formed during the conversion of 9-acridinecarboxaldehyde to acridine (AI) and acridone (AO) by myeloperoxidase within leukocytes were identified. The conversion efficiency of TP in the bloodstream is low, although it is promoted under conditions of UV exposure, chlorine dioxide treatment, and biological treatment [8,42,43]. Therefore, it appears that the detected 9-acridinecarboxaldehyde is primarily generated when it is discharged into rivers during wastewater treatment processes or when it undergoes transformation by ultraviolet radiation in its natural state. 9-Carboxyacridine is a compound that has an acridine structure with a carboxyl group (-COOH) at the ninth position (Table 2). Under oxidative conditions, the hydroxyl groups (–OH) of 10OH-CBZ and DiOH-CBZ are oxidized to ketones (=O), resulting in the formation of 9-carboxyacridine, which has been predicted to be highly genotoxic [6,44].

### 2.3. Spatial and Seasonal Variation in CBZ and Its Transformation Products in Surface Waters of the Mangyeong and Dongjin Rivers

#### 2.3.1. Spatial Variation

**Figure 2:**
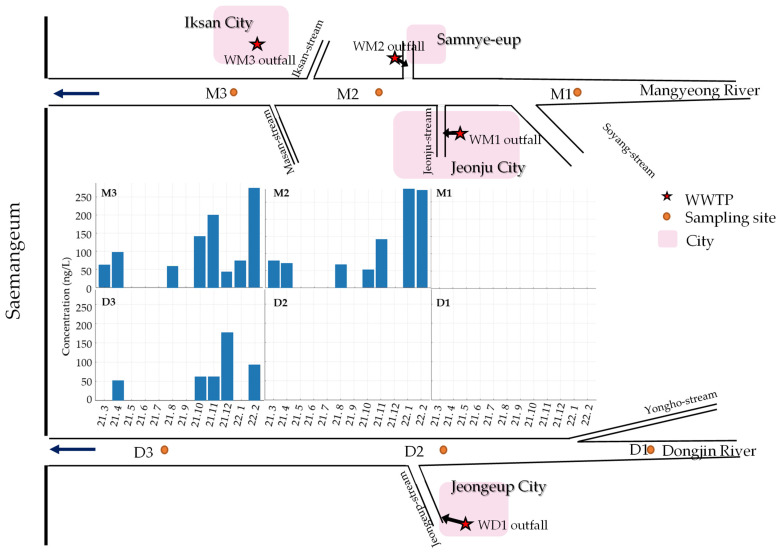
Concentration of CBZ detected in the Mangyeong (**above**) and Dongjin Rivers (**below**).

**Figure 3:**
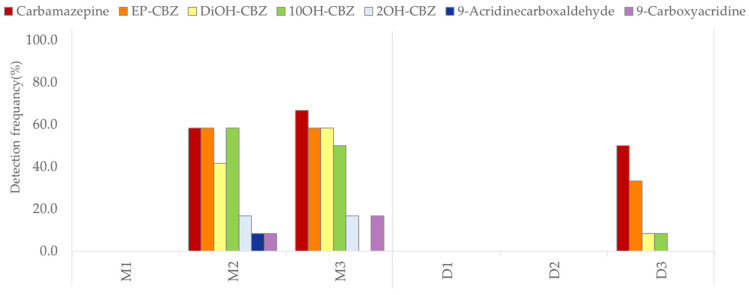
Detection frequency (%) of CBZ metabolites in the Mangyeong and Dongjin Rivers.

The occurrence patterns of chemical substances in surface water are generally influenced by various factors such as Wastewater Treatment Plants (WWTPs), population density, precipitation, and river flow [13,32]. This study compared the spatial distribution of the CBZ and its six TPs (Figure 2 and Figure 3), and detailed information about the WWTPs is provided in Table S3. The Mangyeong River, which passes through the urban areas of Jeonju City (population of 670,411) and Iksan City (population of 280,150), exhibited an approximately 1.4 times higher concentration and 3 times higher detection frequency than the Dongjin River, which passes through an agricultural area with a lower population in Jeongeup City (population of 104,463) (Table 1). The types and detection frequencies of the identified TPs also showed significant differences between regions. Specifically, two additional types of TPs were detected in the Mangyeong River compared to that in the Dongjin River, and their detection frequency was 4.9-fold higher. Furthermore, the concentration of CBZ and the distribution of TPs varied depending on the location of the WWTP being upstream or downstream, demonstrating that the WWTP significantly contributed to the distribution of CBZ and its TPs in both rivers. Prior to the confluence of WWTPs, CBZ and its TPs were not detected upstream; however, a significant increase in the concentration and detection frequency of CBZ was observed at the confluence of WWTPs and the detection frequency of TPs also significantly increased. The detection frequency increased, particularly in the downstream areas of the Mangyeong River, which passes through urban regions. This can be explained by the influence of the WWTP and the confluence of rivers due to the high population density. The Mangyeong River experienced a significant increase in concentration due to the convergence of two wastewater treatment plants (WM1 facility capacity: 403 kton/day; WM2 facility capacity: 32 kton/day) discharging into the M2 point of the river. M3 exhibited changes in concentration due to the inflow from WM3 (a WWTP with a capacity of 100 kton/day) in Iksan City and the Masan Stream. The Dongjin River experienced an increase in the CBZ concentration as it joined WD1 (facility capacity: 58.6 kton/day), which passed through Jeongeup City between D2 and D3; however, the treatment capacity of this WWTP is not high, resulting in relatively low concentrations. These findings indicate that the combination of WWTP and tributary inflow plays a significant role in the concentration of CBZ and the detection frequency of its TPs in the two rivers. The detected TPs included CBZ-EP, DiOH-CBZ, 10OH-CBZ, and 2OH-CBZ, which are the major TPs of CBZ and were found at high concentrations [40,41]. Additionally, 9-acridinecarboxaldehyde and 9-carboxyacridine were also detected (Figure 3, Table 2). Particularly, three TPs (2OH-CBZ, 9-acridinecarboxaldehyde, and 9-carboxyacridine) that were not detected in the Dongjin River were found in the Mangyeong River. This is believed to be due to the high concentration of CBZ and its formation during the wastewater treatment process. 2OH-CBZ is formed through a minor pathway of CBZ, while 9-Acridinecarboxaldehyde is generated more abundantly through UV exposure and chlorine treatment than by the transformation of CBZ within the body. This is attributed to the influence of WM1 and WM2, which employ UV and chlorine treatments for the disinfection of wastewater [8,42,43]. WD1 in the Dongjin River utilizes ultraviolet treatment; however, the facility has an extremely limited capacity, and a low concentration of CBZ is believed to be the cause of non-detection. 9-Carboxyacridine was interpreted as a product generated by the oxidation of DiOH-CBZ, as reported by Miao et al. [14] and Liu et al. [16]. These findings indicate that the low removal efficiency of CBZ in WWTPs is a major factor contributing to the variations in CBZ concentrations in urban wastewater inflows. Additionally, CBZ undergoes various treatment processes (biological and physicochemical) in WWTPs, leading to the formation of different TPs [13,16,45,46,47]. The detection frequencies of the identified TPs in both rivers generally appeared in the following order: CBZ-EP > DiOH-CBZ > 10OH-CBZ > 2OH-CBZ > 9-carboxyacridine > 9-acridinecarboxaldehyde. This indicated a significantly higher detection frequency of major TPs—such as CBZ-EP, DiOH-CBZ—from CBZ (Figure 3) [6,48].

#### 2.3.2. Seasonal Variation

**Figure 4:**
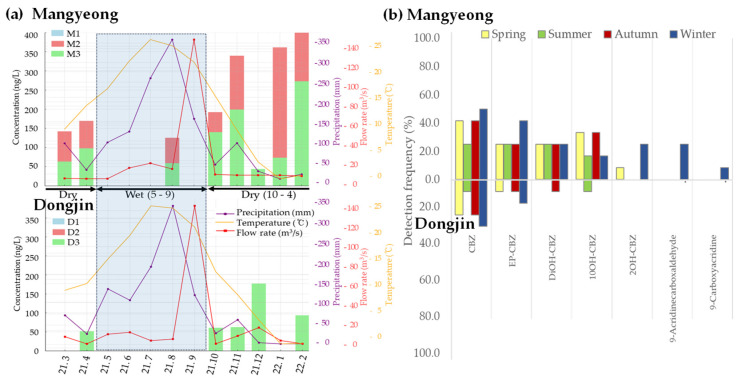
Seasonal variation in CBZ concentration and metabolite detection rate: (**a**) variations in detected concentrations in relation to rainfall (purple), flow rate (red), and temperature (yellow); (**b**) detection frequency of CBZ metabolites in the Mangyeong and Dongjin Rivers.

Figure 4 illustrates the changes in CBZ and TPs over time. Figure 4a shows the difference in CBZ concentrations between the two rivers according to season, in the order of winter > autumn > spring > summer. The CBZ concentration was higher during the dry season than that during the rainy season. Regarding TPs, no significant differences were observed among major TPs. However, the detection frequency was higher during autumn and winter (Figure 4b). This trend was attributed to a decrease in precipitation and streamflow [33]. Korea has a dry season from November to May, and during the dry season, the Mangyeong River showed a concentration difference of approximately 1.9 times compared with that of the Dongjin River (Figure 4). Considering the average flow rate and precipitation of the two rivers, the flow rate of the Mangyeong River during the dry season (November to May) was 5.8 m^3^/s with a precipitation of 50.6 mm, while that of the Dongjin River was 9.2 m^3^/s with a precipitation of 57.8 mm. During the wet season (June–October), the flow rate of the Mangyeong River was 41.4 m^3^/s and the precipitation was 188.0 mm. The flow rate of the Dongjin River was 31.2 m^3^/s and the precipitation was 210.9 mm. Compared with that of the rainy season, the flow rate of the Mangyeong River was reduced to approximately 14% and precipitation was reduced to approximately 25%. Similarly, the flow rate and precipitation of the Dongjin River decreased to approximately 33% [49]. The decrease in flow rate and precipitation led to a reduction in the dilution effect of the CBZ concentration, indicating an increased impact of the WWTP or upstream inflow on water quality [33,50]. In this study, Figure 4a show a sudden decrease in CBZ concentrations in the Mangyeong and Dongjin Rivers during December and January, which, as previously discussed, is likely due to the reduction in flow rate and precipitation, thereby amplifying the relative influence of CBZ concentrations from wastewater treatment plants (WWTP A, WWTP B) and tributary inflows (Iksan and Masan Streams).

### 2.4. Additional Identification of CBZ TPs Based on Generating Molecular Networks

**Figure 5:**
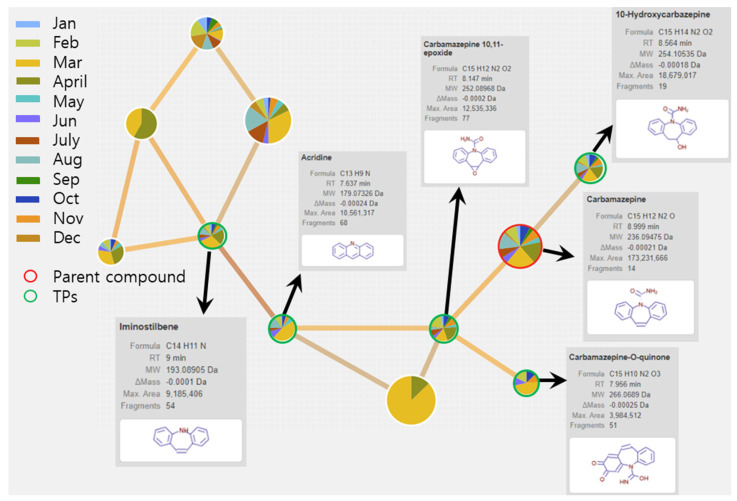
Molecular network for carbamazepine transformation products identification in M3 samples.

Molecular networks were used to identify additional CBZ TPs in samples collected from the M3 site where various TPs were detected. Through this analysis, a cluster consisting of ten nodes, excluding the parent compound CBZ, was identified. The items representing the TPs and structural characteristics of node CBZ are presented in Figure 5. The size of the pie chart represents the area values of the detected TPs, whereas the lines connecting the pie charts represent the associations between the compounds. Although it was difficult to accurately determine seasonal patterns based on the area values of a pie chart, it was possible to confirm consistent detection of the CBZ and its TPs using annual data. In the molecular network centered around CBZ, a clear association was observed with the major TPs (EP-CBZ and 10OH-CBZ) identified in Section 3.3. Through the second and third transformation pathways, iminostilbene (IM) and acridine—which are generated by leukocytes in the body during WWTP (UV/H_2_O_2_) treatment—were identified [16,40,41]. Furthermore, a substance with the structure and name suspected to be a CBZ TP was discovered and confirmed to be CBZ-O-quinone. A literature review revealed that this substance is either 2OH-CBZ, which is generated in the second transformation pathway of CBZ, or TP, which is transformed from 3OH-CBZ or produced in conjunction with EP-CBZ during oxidation. This TP underwent rearrangement, resulting in a decrease in the ring size of the central heterocycle and the formation of 9-acridinecarboxaldehyde. Additionally, it demonstrates an association between EP-CBZ and CBZ-O-quinone within the cluster [51,52].

### 2.5. Proposed Pathway of CBZ Through Non-Targeted Analysis and Molecular Network Analysis

**Figure 6:**
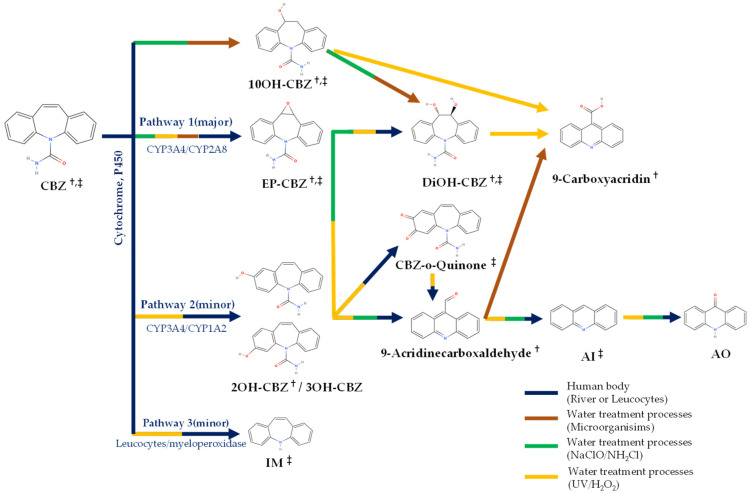
Identification of transformation products and their pathway in surface water through non-targeted analysis and molecular networking. ^†^ Non-target; ^‡^ Generate Molecular networks.

The parent compound CBZ and several TPs were identified in surface water using non-targeted analysis and molecular networking. The TPs detected through non-targeted analysis, excluding 3OH-CBZ and AO as confirmed in previously reported transformation pathways, are presented in Figure 6. As the analysis was limited to surface water samples, the specific origin and transformation pathways of these TPs remain unclear. However, some studies confirmed that CBZ and its TPs can be generated in WWTP not only through human transformation but also through processes such as microbial, chlorine, UV, and hydrogen peroxide treatments [8,14,16,42,43]. In the human body, CBZ is primarily transferred into the liver through the Cytochrome P450 enzyme system. The major pathway (1) is catalyzed by the enzymes CYP3A4/CYP2A8 and converted into various TPs (e.g., EP-CBZ, DiOH-CBZ); the minor pathway (2) is transformed by the enzymes CYP3A4/CYP1A2 (e.g., 2OH-CBZ, 3-OHCBZ); and pathway (3) is transformed by the myeloperoxidase enzyme within white blood cells (IM) [8,40,41]. According to Liu Q et al. [16], CBZ is resistant to degradation; however, it can be converted into various transformation products through diverse treatment processes in WWTPs, such as UV/H_2_O_2_, Na_2_ClO/NH_2_Cl treatments, and microorganisms. Specifically, 9-acridinecarboxaldehyde is known to form more readily through UV exposure and chlorination treatments than through biotransformation within the body, while 10OH-CBZ and AI are also enhanced under UV and chlorine treatment conditions [8,16,42,43]. In this study, the WWTPs at both rivers primarily employed UV and chlorine treatments (Table S3), and the detection of transformation products similar to those reported in previous studies suggests consistency between the observed transformation pathways and the processes used in WWTPs. The CBZ TPs identified through non-targeted analysis and molecular networking provided crucial information for understanding the complex transformation pathways and environmental transformations of CBZ. These results can contribute to the effective identification of various TPs that are difficult to identify through targeted analysis alone and by tracking their transformation pathways. Furthermore, the findings of this study contribute to a better understanding of the causes and impacts of CBZ concentration fluctuations in specific seasons and regions. This information could be used as crucial data for future WWTP and environmental monitoring programs.

## 3. Materials and Methods

### 3.1. Chemical Standards and Reagents

Carbamazepine reference standard was purchased from Sigma-Aldrich (The Woodlands, TX, USA), and isotope-labeled carbamazepine-D10 (98%) internal standard was purchased from Cambridge Isotope Laboratories (catalog number DLM-2806-1.2; Andover, MA, USA). Standards stock solutions were prepared in acetonitrile at a concentration of 100 mg/L and stored at −20 °C in the dark. Formic acid (≥99.0%) and HPLC MS grade of methanol, deionized water, and acetonitrile were purchased from Fisher Scientific (Loughborough, UK). Ammonium formate (97% purity) was purchased from Sigma-Aldrich (St. Louis, MO, USA). A 0.20-micrometer mixed cellulose–ester sterilized membrane filter with a 25-millimeter diameter (ADVANTEC, Higashiosaka, Japan) was used for pre-treatment.

### 3.2. Sampling Sites and Sample Collection

**Figure 7:**
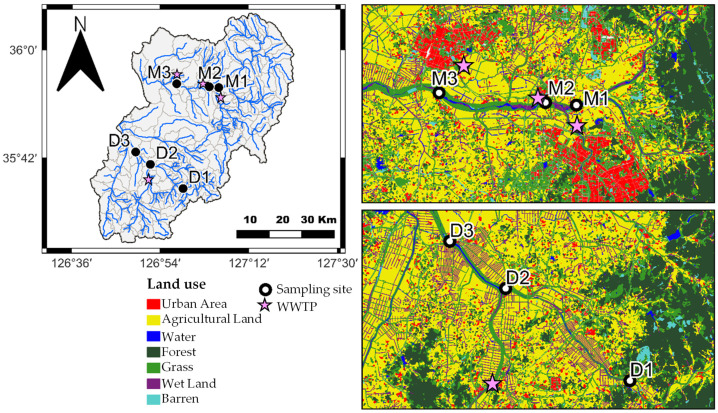
Map of the study area: sampling sites’ distribution along the Mangyeong and Dongjin Rivers.

The Saemangeum Watershed encompasses the Mangyeong (M) and Dongjin (D) Rivers and is characterized by diverse land uses, including agriculture, forestry, and urban areas. Target locations were selected based on the position of the WWTPs, and monitoring was conducted by dividing them into urban and agricultural areas to assess spatial changes. The Mangyeong River Basin is primarily composed of urban areas, passing through Jeonju (population of 670,411) and Iksan (population of 280,150) Cities, and includes the Jeonju and Iksan Streams. In this area, three WWTPs are present (WM1, WM2, and WM3 with processing capacities of 403 kton/day, 32 kton/day, and 100 kton/day, respectively). The Dongjin River Basin passes through an agricultural region that produces approximately 234,000 tons of rice annually, including Jeongeup City (population of 104,463) and a WWTP (WD1: processing capacity of 58.6 kton/day), as reported by the Ministry of the Statistics Korea [53] and Ministry of Environment [54]. Surface water samples were collected from three locations within the catchment areas of the Mangyeong and Dongjin Rivers. The sampling was conducted monthly from March 2021 to February 2022. The geographical locations of the sampling points and WWTPs are shown in Figure 7. The sampling points of the Mangyeong River consisted of an upstream point (M1), a midstream point (M2) that passed through Jeonju City, and a downstream point (M3) that passed through Iksan City and included the Iksan and Masan Streams. The sampling points of the Dongjin River consisted of the uppermost point (D1), middle point (D2), and downstream point (D3), which included the Jeongeup Stream. The total number of samples was 72 (6 locations, 12 months, and 1 sample per location). The collected water samples were filtered on site using a 0.20-micrometer mixed cellulose–ester sterile membrane filter. The filtered samples were then transported to the laboratory in a cooler to maintain low temperatures. Prior to analysis, the samples were stored at −20 °C in a freezer. After thawing, the samples were centrifuged at 2800 rpm for 10 min, and only the supernatant was used for analysis.

### 3.3. Liquid Chromatography (LC)–Orbitrap/High-Resolution Mass Spectrometry Analysis

Sample preprocessing and analysis were performed using an Ultimate 3000 UHPLC (Thermo Scientific, Waltham, MA, USA) equipped with an EQuan MAX online SPE (Thermo Fisher Scientific, Waltham, MA, USA) automated sample preprocessor coupled with a Q Exactive Plus Orbitrap mass spectrometer (Thermo Fisher Scientific, San Jose, CA, USA). The purification and concentration of the samples were performed using Hypersil GOLD aQ online solid-phase extraction (online SPE) columns (20 × 2.1 mm and 12 μm particle size; Thermo Scientific, Vilnius, Lithuania). Furthermore, the separation of CBZ and non-targeted compounds was achieved using a reverse-phase CORTECS T3 analytical column (100 × 2.1 mm and 1.6 μm particle size; Waters, Milford, CT, USA). Additionally, mobile-phase eluents (A: water with 0.1% (*v*/*v*) formic acid and 5 mM ammonium formate; B: methanol with 0.1% (*v*/*v*) formic acid and 5 mM ammonium formate) were used. One thousand microliters of the sample was injected into the loop using an autosampler with an online SPE column. The mobile and separable column operating conditions and equipment analysis conditions for Online SPE are detailed in Table S1.

The ionization mode was positive when a heated electrospray ionization (HESI) source was used. This operated under the following conditions: sheath gas, 40; auxiliary gas, 1.0 a.u; sweep gas flow rate, 2; spray voltage, 3800 V; capillary temperature and auxiliary gas temperature, both 330 °C; S-lens RF level, 50.0. The quadrupole-Orbitrap mass spectrometer was operated under the following conditions: full-scan MS data-dependent MS^2^ (full scan/ddMS^2^) mode, suitable for non-targeted compound analysis. Full scan MS mass range, 100–1500 *m*/*z*; resolution, 70,000; automatic gain control (AGC), 1.0 × 10^6^; maximum injection time, 100 ms; ddMS^2^ resolution, 17,500; AGC, 3.0 × 10^6^; maximum injection time, 50; collision energy-stepped, 15, 30, 50 V; mass error, ±5 ppm. Automatically, through the full scan/ddMS^2^, MS/MS data collection was possible for ten precursor ions, depending on the conditions, in the order of sensitivity at the same retention time.

### 3.4. Quality Assurance and Control

The analytical method was validated by evaluating linearity (r^2^), accuracy, and precision. Linearity was assessed using a fixed concentration of 50 μg/L for the internal standard (IS) and carbamazepine working solutions ranging from 10 to 200 μg/L, which were diluted in distilled water to achieve final concentrations of 50 to 2000 ng/L. The LOQ was determined by analyzing a concentration of 100 ng/L in seven replicates and multiplying the standard deviation by 10. Accuracy and precision were evaluated by analyzing a concentration of 200 ng/L in seven replicates (Table S2).

### 3.5. Data Processing for Non-Targeted Compound Identification

**Figure 8:**
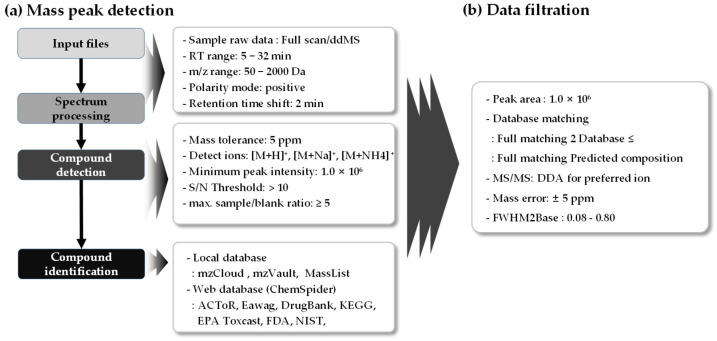
Workflow for non-target analysis: (**a**) mass peak detection and (**b**) data filtration.

The raw data were processed using Compound Discoverer 3.3 (Thermo Fisher Scientific Inc.). Non-targeted analysis was performed by modifying the “Environmental Unknown ID database and the molecular network” workflow provided in the software. The data processing conditions were as follows: mass tolerance ≤ 5 ppm; detected ions, positive [M + H]^+^, [M + Na]^+^, [M + NH^4^]^+^; minimum peak intensity ≥ 1.0×10^6^; S/N threshold > 10; max. sample/blank ratio ≥ 5 a.u.; local databases (mzCloud, mzVault, and MassList); and web databases (ChemSpider: ACTOR, DrugBank, Eawag, EPA Toxcast, FDA, KEGG, NIST). The “fill the gap” node in this workflow is a setting that automatically fills in the gap of the area of a substance if its concentration detected is lower than the ILOD used. Due to the potential confusion caused by falsely detected substances, this node was deemed unsuitable and therefore excluded. After peak detection, the identification process for selecting CBZ TPs was set according to the method proposed by Im et al. [13] to increase reliability. This method categorizes the confidence levels of the generated data from the lowest, Level 5, to that of the highest, Level 1, which is used when setting the criteria [39]. The identification process was conducted by confirming the isotope pattern, MS spectrum, MS^2^ spectrum, among others, and if the minimum requirements are met at each level, the corresponding level can be trusted. A higher level indicates greater reliability and Level 1 corresponds to target analysis using standard substances. If no standard substance was present, grading was determined using MS and MS^2^. In non-targeted analysis, Level 2 confidence was assigned when a specific pattern of MS^2^ was detected or when a match was confirmed with reliable libraries such as mzCloud, mzVolt, and MassBank. Level 3 was assigned for agreement between the MS^2^ information that can be generated from the predicted structure of the detected substance in the absence of comparable MS^2^ information and the actual analyzed MS^2^. They can be classified into Level 4 (matching MS, isotopic pattern, and adduct information) and 5 (MS). The minimum amount of information required for each level is listed in Table 3. The data filtering process was applied to the dataset obtained from the previous mass-peak detection process to achieve a confidence level of Level 2. The filtering conditions were specified as follows, based on the criteria used by Gonzalez-Gaya et al. [55] and Lopez-Herguedas et al. [56]: minimum peak intensity ≥ 1.0 × 10^6^; mass tolerance ≤ 5 ppm; MS^2^ pattern matching; exclusion of peaks detected in the background sample, and inclusion of 2 or more matches from online/offline databases (including the predicted composition). Detailed information is presented in Figure 8.

The generation of Molecular Networks nodes was conducted by adding the “Generate Molecular Networks node” in the same software. The settings were configured in the same manner as before with some modifications to the default values. To observe all transformation processes, phase I (dehydration, hydration, etc.) and phase II (acetylation, arginine conjugation, etc.) were selected and performed for a mass tolerance of 2.5 ppm or higher. The extracted items were used to form a network, selecting only compounds with a confidence level of two or higher, as determined by the data filtration mentioned above.

## 4. Conclusions

This study investigated the detection characteristics of CBZ in the Mangyeong and Dongjin Rivers and tracked its transformation pathway using NTS. The concentration of CBZ varies depending on the region and season, with the Mangyeong River passing through urban areas and exhibiting high levels during the dry season. In the NTA results, three types of transformation products (DiOH-CBZ, EP-CBZ, and 10OH-CBZ) were detected in both rivers. Three transformation products (2OH-CBZ, 9-acridinecarboxaldehyde, and 9-carboxyacridine) were identified in the Mangyeong River. Through analysis using GMN, we confirmed the presence of TPs (CBZ-O-quinone, iminostilbene, and acridine) that were not detected by NTA. Based on this, we propose a transformation pathway for CBZ. This study provides insights into the variations in CBZ concentration according to season and region as well as the distribution, origin, and transformation pathways of its TPs. This helped us understand the causes of these variations and their impacts. This information can be used as crucial data for future WWTPs and environmental monitoring programs.

## Figures and Tables

**Table 1:** Concentrations of CBZ in Mangyeong and Dongjin Rivers.

Site		Concentration (ng/L)			Detection Frequency DF (%)
		Range	Mean	Median	
Mangyeong River	M1 (n = 12)	<LOQ	<LOQ	<LOQ	
	M2 (n = 12)	52.2–288.2	140.5	79.1	58.3
	M3 (n = 12)	43.8–273.1	118.6	85.6	66.7
Total (n = 36)		43.8–288.2	128.8	79.1	41.7
Dongjin River	D1 (n = 12)	n/d	n/d	n/d	-
	D2 (n = 12)	<LOQ	<LOQ	<LOQ	-
	D3 (n = 12)	51.7–177.7	89.0	62.4	50
Total (n = 36)		51.7–177.7	89.0	62.4	13.9

n/d: not detected, n: number of samples.

**Table 2:** Non-targeted list of compounds identified in samples with various parameters (retention time (RT), molecular formula, *m*/*z*, mass accuracy, match score, confidence level, and structure).

No.	Name	RT (min)	Formula	*m*/*z*	Mass Accuracy (ppm)	Match Score	Confidence Level	Structure
1	EP-CBZ	7.666	C_15_H_12_N_2_O_2_	253.097	−0.47	96.4	2a ^†,‡^	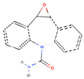
2	DiOH-CBZ	7.670	C_15_H_14_N_2_O_3_	271.108	−0.9	73.2	2a ^†,‡^	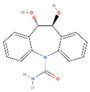
3	2OH-CBZ	7.872	C_15_H_12_N_2_O_2_	253.097	−0.81	-	2b ^†,‡^	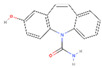
4	10OH-CBZ	7.870	C_15_H_14_N_2_O_2_	255.113	−0.96	96.7	2a ^†,‡^	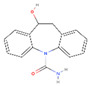
5	9-Acridinecarboxaldehyde (9-Formylacridne)	8.576	C_14_H_9_NO	208.076	−0.81	-	2b ^†^	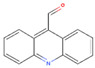
6	9-Carboxyacridine (9-Acridinecarboxylic acid)	8.776	C_14_H_9_NO_2_	224.070	−2.97	84	2a ^†^	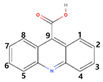

Level 2a: MS^2^ spectrum matching of mzCloud, Level 2b: MS^2^ spectrum matching—online DB. ^†^ Mangyeong River. ^‡^ Dongjin River.

**Table 3:** Proposed identification confidence tiers in high-resolution mass spectrometry.

Identification Confidence			Minimum Data Requirements
Level 1:	confirmed structure by reference standard		MS/MS^2/^RT/Reference Std.
Level 2:	Level 2a:	probable structure by library spectrum match	MS/MS^2/^Library(in house DB) MS^2^
	Level 2b:		MS/MS^2^/Library(online DB) MS^2^
Level 3:	tentative candidate by structure, substituent, class		MS/MS^2/^Exp. Data
Level 4:	unequivocal molecular formula		MS/Isotope/adduct
Level 5:	exact mass of interest		MS

## Data Availability

Data is contained within the article.

## Supplementary Materials

The following supporting information can be downloaded at: https://www.mdpi.com/article/10.3390/ijms252211947/s1.
