# Supplementary material for: Non-Targeted Screening and Identification of the Transformation Pathway of Carbamazepine in the Saemangeum Watershed, Republic of Korea
Source: Int J Mol Sci. 2024 Nov 7;25(22):11947. doi: 10.3390/ijms252211947 (PMC11593937; doi:10.3390/ijms252211947)
Supplement: Supplementary file 1 [file ijms-25-11947-s001.zip › ijms-3247218-supplementary.pdf]

Supplementary Data

Non-Targeted Screening and Identification of the Transformation Pathway of Carbamazepine in the Saemangeum Watershed, Republic of Korea

Da Rae Jeon, Young-Eun Kim, Jong Kwon Im, Yujeong Huh and Hyoung Seop Kim *

Environmental Measurement & Analysis Center, National Institute of Environmental Research, 42 Hwangyong-ro, Incheon 22689, Republic of Korea; ekfo1228@korea.kr (D.R.J.); happyday23@korea.kr (Y.-E.K.); lim-jkjk@daum.net (J.K.I.); huhyujeong@korea.kr (Y.H.)

* Correspondence: hsupkim@korea.kr

Figure S1. Overview of compound identification workflow by Compound Discoverer3.3 showing the Level 2 identification of CBZ TPs in sample as an example (a) MS spectrum (b) Level 2b: MS2 spectrum matching – Other DB (c) Level 2a: MS2 spectrum matching– mzCloud

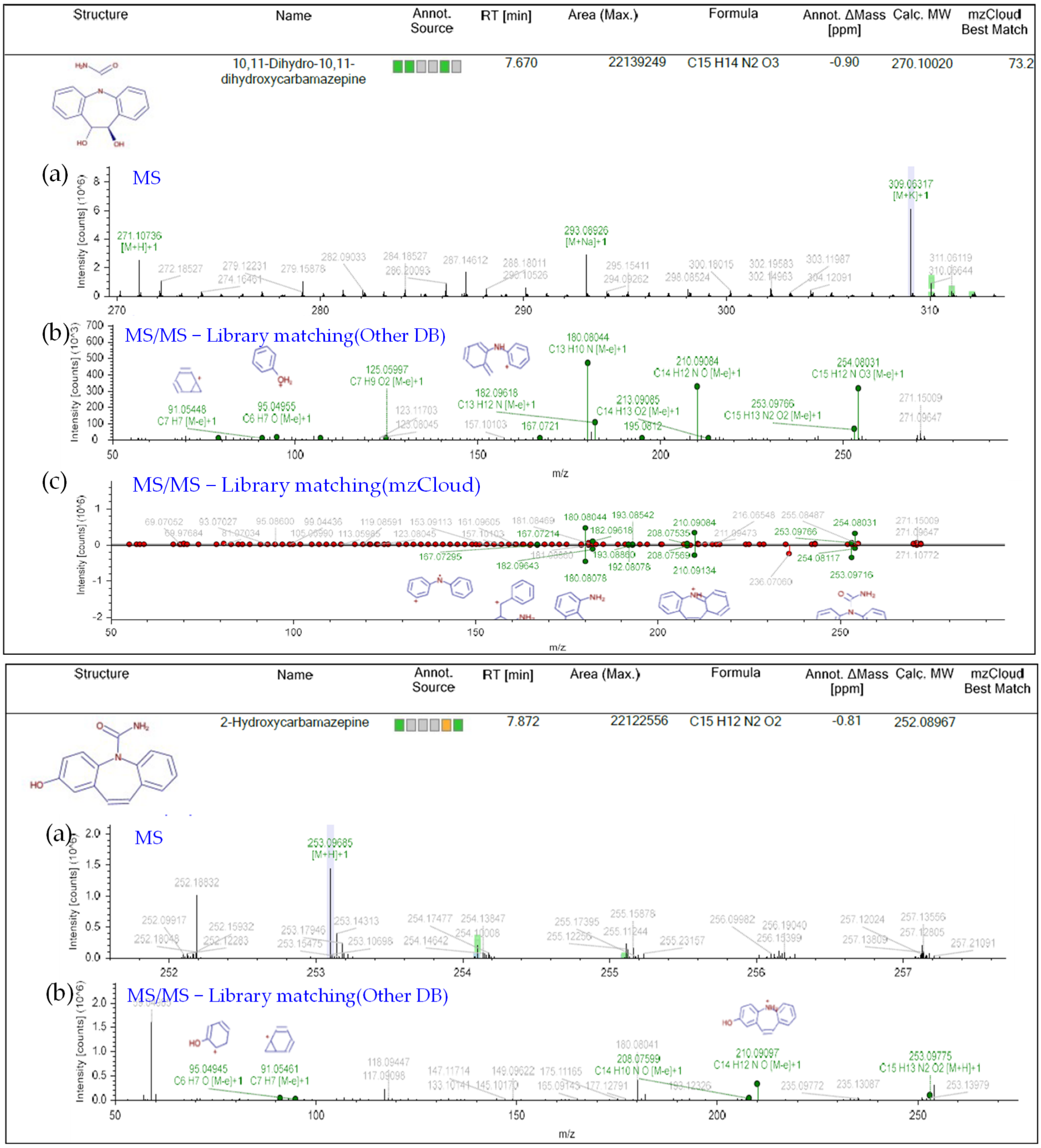

Structure	Name	Annot. Source	RT [min]	Area (Max.)	Formula	Annot. ΔMass [ppm]	Calc. MW	mzCloud Best Match
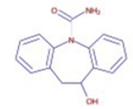	10-Hydroxycarbazepine		7.870	15895308	C15 H14 N2 O2	-0.96	254.10528	96.7

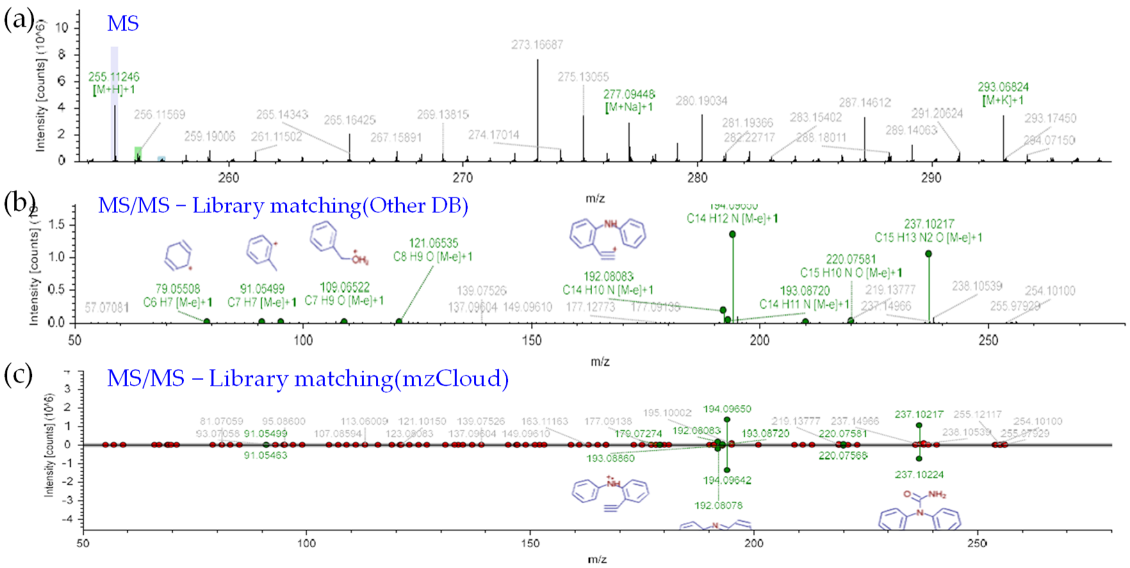

Structure	Name	Annot. Source	RT [min]	Area (Max.)	Formula	Annot. ΔMass [ppm]	Calc. MW	mzCloud Best Match
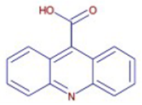	9-acridinecarboxylic acid hydrate		8.776	12064813	C14 H9 N O2	-2.97	223.06267	84.0

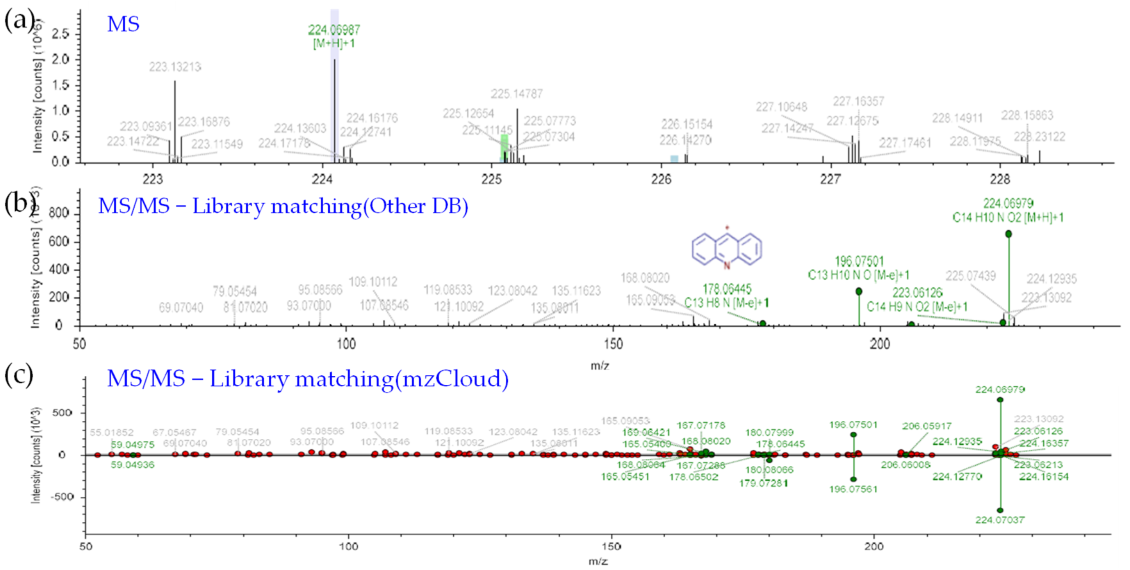

Structure	Name	Annot. Source	RT [min]	Area (Max.)	Formula	Annot. ΔMass [ppm]	Calc. MW	mzCloud Best Match
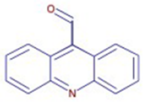	9-Formylacridine		8.576	11540396	C14 H9 N O	-0.81	207.06825	

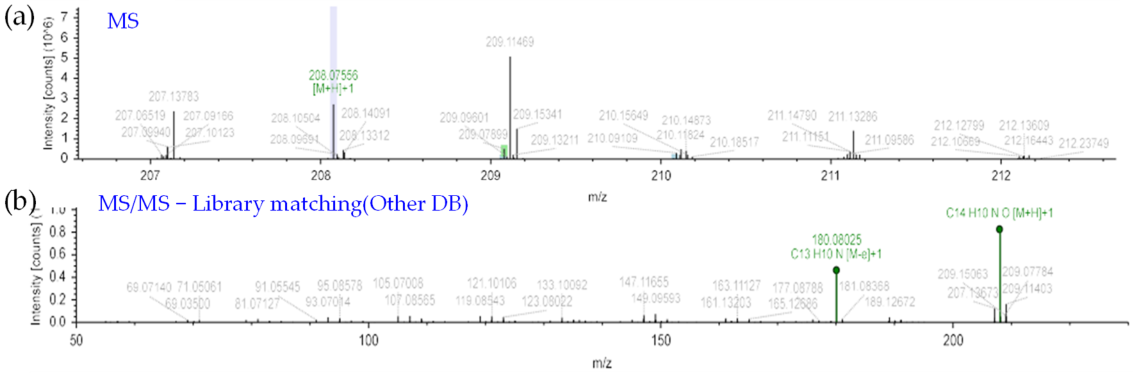

Table S1. On-line SPE & LC parameters

<table>
<tr><td colspan="7">Dionex Ultimate 3000-Q</td></tr>
<tr><td>Instrument</td><td colspan="3">LC parameters</td><td colspan="3">On-line SPE parameters</td></tr>
<tr><td>Column</td><td colspan="3">CORTECS T3
(100 × 2.1mm, 1.6 μm)</td><td colspan="3">Hypersil GOLD aQ
(20 × 2.1 mm, 12 μm)</td></tr>
<tr><td>Mobile phase</td><td colspan="6">A: Water (0.1% Formic acid, 5mM Ammonium formate)
B: Methanol (0.1% Formic acid, 5mM Ammonium formate)</td></tr>
<tr><td>Pump</td><td colspan="3">Pump2 (HPG-3400RS)</td><td colspan="3">Pump 1 (LPG-3400SD)</td></tr>
<tr><td rowspan="9">Gradient</td><td>Retention (min)</td><td>%B</td><td>Flow (mL/min)</td><td>Retention (min)</td><td>%B</td><td>Flow (mL/min)</td></tr>
<tr><td>0</td><td>5</td><td>0.2</td><td>0</td><td>2</td><td>1.0</td></tr>
<tr><td>1.1</td><td>5</td><td>0.2</td><td>1.0</td><td>2</td><td>1.0</td></tr>
<tr><td>5.0</td><td>70</td><td>0.2</td><td>1.1</td><td>2</td><td>0.05</td></tr>
<tr><td>7.0</td><td>70</td><td>0.2</td><td>27.1</td><td>2</td><td>0.05</td></tr>
<tr><td>22.0</td><td>100</td><td>0.2</td><td>28.0</td><td>2</td><td>1.0</td></tr>
<tr><td>27.0</td><td>100</td><td>0.2</td><td>32.0</td><td>2</td><td>1.0</td></tr>
<tr><td>28.0</td><td>5</td><td>0.2</td><td colspan="3" rowspan="2"></td></tr>
<tr><td>32.0</td><td>5</td><td>0.2</td></tr>
<tr><td rowspan="2">Column temperature</td><td colspan="3" rowspan="2">40 ℃</td><td colspan="2">Injection volume</td><td>1000 μL</td></tr>
<tr><td colspan="2">Purification</td><td>2% MeOH</td></tr>
</table>

Table S2. QA/QC results for carbamazepine compound

Compound	Formular	Precursor ion (Da)	Retention time (min)	Adduct	r^2	LOQ (ng/L)	Accuracy	Precision (n=7)
Carbamazepine	C15H12N2O	237.10224	8.97	M+H	0.9993	40.6	100.7	2.5

Table S3. Information of the surveyed WWTPs

WWTP	River	Operation capacity (m3/day)	Treatment process	Effluent disinfection
Jeonju wastewater treatment plant (WM1)		403,000	CNR, CSBR	Cl, UV
Wanju industrial complex wastewater treatment plant (WM2)	Mangyeong	32,000	MLE	Cl, UV
Iksan wastewater treatment plant (WM3)		100,000	MLE	UV
Jeongeup wastewater treatment plant (WD1)	Dongjin	58,600	AO	Cl, UV

CNR: Comlete nitrification reactor, CSBR: Cyclic sequencing batch reactor, MLE: Modified Ludzack-Ettinger process, AO: Anaerobic oxic process, Cl: Chlorination, UV: Ultraviolet disinfection
